# Supplementary figures and images for: Belly roll, a GPI-anchored Ly6 protein, regulates Drosophila melanogaster escape behaviors by modulating the excitability of nociceptive peptidergic interneurons
Source: eLife. 2023 Jun 13;12:e83856. doi: 10.7554/eLife.83856 (PMC10264074; doi:10.7554/eLife.83856)

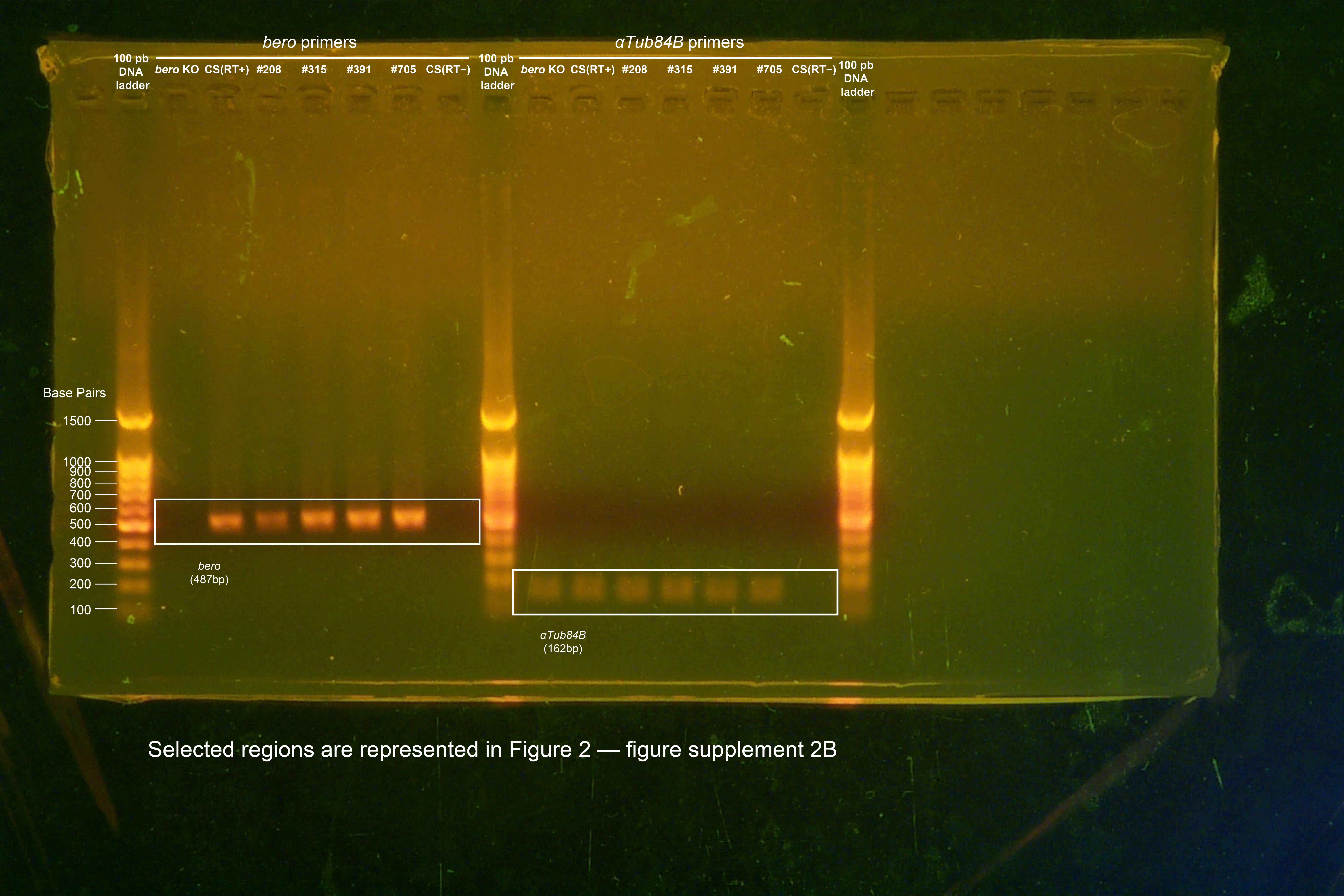

Supplement: Figure 2—figure supplement 2—source data 2. [file elife-83856-fig2-figsupp2-data2.zip › Figure 2-figure supplement 2-Source data 2/Labelled figure with the uncropped gel.png]

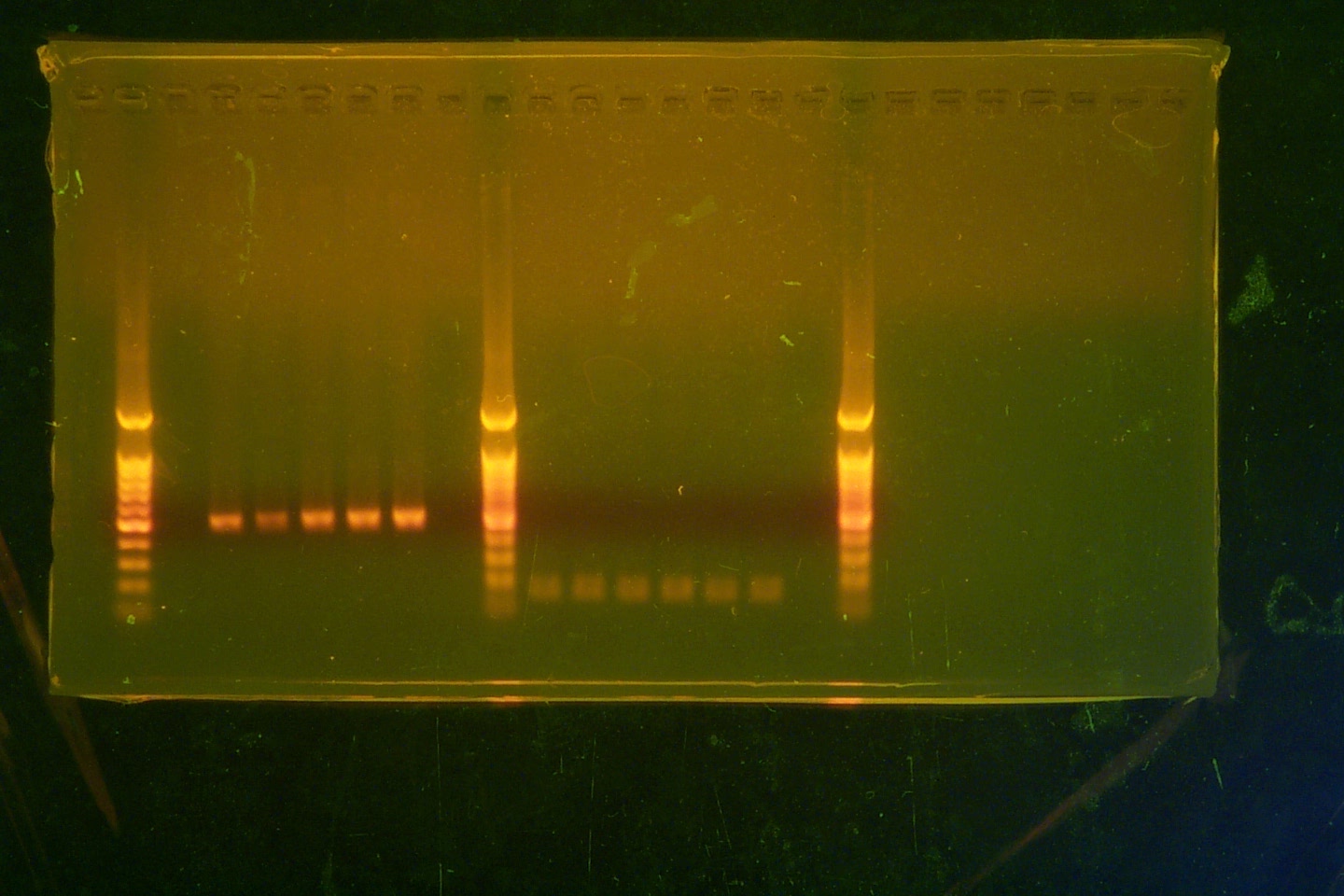

Supplement: Figure 2—figure supplement 2—source data 2. [file elife-83856-fig2-figsupp2-data2.zip › Figure 2-figure supplement 2-Source data 2/Full raw unedited gel.JPG]
